# NSUN4 Suppresses Ferroptosis Through m^5^C-Dependent Stabilization of C-MYC and Activation of the PI3K/Akt Signaling Pathway in Cervical Cancer

**DOI:** 10.3390/cancers18091392

**Published:** 2026-04-28

**Authors:** Duancheng Tian, Ming Du, Zhen Zheng, Weidi Wang, Haoyu Wang, Reyilanmu Maisaidi, Yang Xiang

**Affiliations:** 1 Department of Obstetrics and Gynecology, Peking Union Medical College Hospital, Chinese Academy of Medical Sciences and Peking Union Medical College, Beijing 100730, China; s2023001015@pumc.edu.cn (D.T.); b2023001104@pumc.edu.cn (M.D.); b2024001116@student.pumc.edu.cn (Z.Z.); b2024001267@student.pumc.edu.cn (W.W.); wanghy@student.pumc.edu.cn (H.W.); b2025001053@student.pumc.edu.cn (R.M.); 2 Department of Obstetrics and Gynecology, National Clinical Research Center for Women’s Health and Obstetric and Gynecologic Diseases, Beijing 100730, China

**Keywords:** cervical cancer, Ferroptosis, RNA m^5^C methylation

## Abstract

Objectives: This study aimed to investigate the biological role and molecular mechanism of the RNA m^5^C methyltransferase NSUN4 in cervical cancer progression, with a focus on its involvement in ferroptosis regulation. Methods: Differential expression and survival analyses were performed using TCGA and GEPIA datasets. Functional enrichment and GSEA identified pathways associated with NSUN4 dysregulation. NSUN4 expression was validated in clinical tissues by qRT-PCR, Western blot, and immunohistochemistry. Gain- and loss-of-function assays, including CCK-8, colony formation, and Transwell assays, were conducted to assess cell proliferation and invasion. Furthermore, a nude mouse subcutaneous xenograft model was established to validate the oncogenic role of NSUN4 in vivo. Ferroptosis was evaluated using specific inhibitors and measurement of GSH and ferroptosis-related proteins. RIP, m^5^C-RIP, RNA stability, and dual-luciferase assays were performed to explore the underlying mechanism. Results: NSUN4 was markedly upregulated in cervical cancer tissues and correlated with poor prognosis. Functionally, NSUN4 enhanced tumor cell growth, migration, and invasion while inhibiting ferroptosis. Mechanistically, NSUN4 bound to and stabilized C-MYC mRNA via m^5^C methylation, activating the PI3K/Akt signaling pathway and promoting ferroptosis resistance. Conclusions: NSUN4 promotes cervical cancer progression by stabilizing C-MYC mRNA through m^5^C modification, leading to PI3K/Akt activation and suppression of ferroptosis. These findings identify NSUN4 as a novel oncogenic regulator and potential therapeutic target in cervical cancer.

## Simple Summary

Cervical cancer remains a major global health challenge; understanding the underlying mechanisms of cancer cell survival and malignant progression is crucial for developing more effective treatments. This study investigated the critical role of the RNA methyltransferase NSUN4 in cervical cancer progression. Our primary objective was to elucidate how NSUN4 stabilizes C-MYC in an m^5^C-dependent manner, thereby activating the PI3K/Akt signaling pathway and ultimately suppressing ferroptosis in cervical cancer cells. We discovered that NSUN4 expression is abnormally elevated in cervical tumors, significantly promoting tumor growth and resistance to cell death. Specifically, NSUN4 modifies and stabilizes the key oncogene C-MYC via m^5^C methylation to activate the PI3K/Akt pathway. These findings provide an important basis for a deeper understanding of cervical cancer biology. Consequently, this study establishes NSUN4 as a key driver of cervical cancer, offering a highly promising novel target for future targeted therapies and drug development.

## 1. Introduction

Cervical cancer remains one of the most common malignancies affecting women worldwide, ranking as the fourth leading cause of cancer-related death among females [1,2]. Despite advances in screening and vaccination programs, the incidence and mortality rates of cervical cancer remain high in developing countries [3]. Although early-stage disease can be effectively managed with surgery or radiochemotherapy, patients with recurrent or metastatic lesions often exhibit poor therapeutic response and unfavorable prognosis [4,5]. The limited efficacy of current treatments underscores the urgent need to uncover the molecular mechanisms driving cervical cancer progression and to identify new therapeutic targets [6,7]. Epigenetic RNA modifications have emerged as critical regulators of tumor biology [8,9]. Among these, 5-methylcytosine (m^5^C) methylation, catalyzed by the NSUN family of RNA methyltransferases, has been implicated in various aspects of RNA metabolism, including stability, translation, and export [10,11]. Dysregulated m^5^C modification contributes to oncogenesis, yet its precise role in cervical cancer remains largely unexplored [12]. NSUN4, originally characterized as a mitochondrial rRNA methyltransferase, has been increasingly recognized to methylate cytoplasmic mRNA targets, influencing tumor growth and drug resistance [13,14,15]. However, its biological functions and regulatory networks in cervical cancer have not been systematically investigated. Ferroptosis, a recently defined iron-dependent form of regulated cell death driven by lipid peroxidation, has attracted growing attention as a novel vulnerability in cancer therapy [16,17]. Unlike apoptosis or necrosis, ferroptosis is determined by the balance between oxidative stress and antioxidant defense systems, primarily governed by the glutathione (GSH)–GPX4 axis [18]. Mounting evidence indicates that tumor cells often acquire ferroptosis resistance to maintain survival under metabolic stress [19]. Several oncogenic signaling pathways, including PI3K/Akt, have been shown to protect cells from ferroptosis by upregulating antioxidant capacity and lipid remodeling [20]. However, how epitranscriptomic regulation via m^5^C methylation affects ferroptosis in cervical cancer is still poorly understood. In this study, we identified NSUN4 as a novel ferroptosis suppressor and oncogenic driver in cervical cancer. Transcriptomic and functional analyses demonstrated that NSUN4 expression is markedly elevated in tumor tissues and correlates with poor prognosis. Mechanistically, NSUN4 directly binds to and stabilizes C-MYC mRNA through m^5^C modification, thereby activating the PI3K/Akt signaling pathway and enhancing cellular antioxidant capacity to suppress ferroptosis. Our findings reveal a previously unrecognized NSUN4–C-MYC–PI3K/Akt axis that links RNA methylation to ferroptosis regulation and tumor progression, providing new insights into the molecular pathogenesis of cervical cancer and potential strategies for targeted therapy.

## 2. Materials and Methods

### 2.1. Data Collection and Bioinformatics Analysis

RNA-sequencing data and corresponding clinical information for cervical squamous cell carcinoma and endocervical adenocarcinoma (TCGA-CESC) were retrieved from The Cancer Genome Atlas (TCGA, https://portal.gdc.cancer.gov/, accessed on 1 January 2025). A total of 305 tumor samples and 3 normal samples were included in the differential expression analysis. Differentially expressed genes were identified using the “limma” package in R (v4.2.2) with an adjusted *p* < 0.05 and |log_2_FC| > 1 as cutoff values. The prognostic value of NSUN4 was assessed via the GEPIA web server (http://gepia.cancer-pku.cn/). Functional annotation, including Gene Ontology (GO), Kyoto Encyclopedia of Genes and Genomes (KEGG), and Gene Set Enrichment Analysis (GSEA), was carried out using the “clusterProfiler” package. Enrichment terms were considered significant at an adjusted *p* < 0.05.

### 2.2. Clinical Specimens

Paired cervical cancer and adjacent noncancerous tissues were collected from patients undergoing primary surgery at Peking Union Medical College Hospital (PUMCH) between April and December 2025. None of the enrolled patients had received chemotherapy or radiotherapy before operation. Adjacent noncancerous tissues were obtained at least 3 cm away from the tumor margin and were confirmed to be free of tumor cells by histopathological examination. All samples were confirmed by histopathology, snap-frozen in liquid nitrogen, and stored at −80 °C. Written informed consent was obtained from each participant, and the study protocol was approved by the PUMCH Ethics Committee.

### 2.3. Cell Culture and Transfection

Human cervical cancer cell lines (HeLa and SiHa) were purchased from the Cell Bank of the Chinese Academy of Sciences (Shanghai, China). Cells were cultured in DMEM (Gibco, Grand Island, NY, USA) supplemented with 10% fetal bovine serum and 1% penicillin–streptomycin under 37 °C and 5% CO_2_.

NSUN4 overexpression plasmids and small interfering RNAs (siRNAs) were synthesized by GenePharma (Shanghai, China). Transfections were performed using Lipofectamine 2000 (Invitrogen, Carlsbad, CA, USA) according to the manufacturer’s protocol. Transfection efficiency was verified 48 h later by qRT-PCR and Western blot. Pre-packaged lentiviral particles expressing shRNA targeting NSUN4 (sh-NSUN4) and a negative control (sh-NC) were purchased from GenePharma (Shanghai, China). HeLa cells were infected with the lentivirus at a multiplicity of infection (MOI) of 5 in the presence of 8 µg/mL polybrene. After 48 h of infection, cells were cultured in a medium containing 2 µg/mL puromycin for 2 weeks to obtain stable clones.

### 2.4. Quantitative Real-Time PCR (qRT-PCR)

Total RNA was isolated using TRIzol reagent (Thermo Fisher Scientific, Waltham, MA, USA). All RNA samples were further treated prior to reverse transcription with the gDNA Eraser reaction included in the PrimeScript™ RT Reagent Kit with gDNA Eraser (Perfect Real Time) (Takara, Kusatsu, Shiga, Japan) to eliminate residual genomic DNA contamination. According to the manufacturer’s instructions, genomic DNA elimination was first performed in a 10 μL reaction containing 2.0 μL 5× gDNA Eraser Buffer, 1.0 μL gDNA Eraser, 1000 ng total RNA, and RNase-free dH_2_O to the final volume. The reaction was incubated at 42 °C for 2 min and then placed on ice. Reverse transcription was subsequently performed in a total volume of 20 μL by adding 10.0 μL of the genomic DNA elimination reaction mixture to 4.0 μL 5× PrimeScript Buffer 2 (for Real Time), 1.0 μL PrimeScript RT Enzyme Mix I, 1.0 μL RT Primer Mix, and 4.0 μL RNase-free dH_2_O. The reverse transcription program consisted of 37 °C for 15 min, followed by 85 °C for 5 s, and then held at 4 °C. The reverse transcription program was 37 °C for 15 min, followed by 85 °C for 5 s, and then held at 4 °C. The resulting cDNA was diluted 5- to 10-fold before qRT-PCR. qRT-PCR was performed using Power SYBR Green Master Mix (Applied Biosystems, Foster City, CA, USA) on a QuantStudio 1 Real-Time PCR System. Each reaction was carried out in a total volume of 10 μL, containing 5.0 μL 2× Power SYBR Green Master Mix, 0.2–0.4 μL each of forward and reverse primers (10 μM), 1.0–2.0 μL diluted cDNA template, and nuclease-free water to the final volume. The amplification program consisted of an initial denaturation at 95 °C for 10 min, followed by 40 cycles of 95 °C for 15 s and 60 °C for 1 min. Melting curve analysis was performed after amplification to verify product specificity. ACTB was used as the endogenous control. Relative expression levels were calculated using the 2^−ΔΔCt^ method. Each sample was analyzed in triplicate. No-template controls (NTC), no-reverse-transcription controls (No-RT), and positive controls were included in each run. Only reactions showing a single specific melting peak were considered valid for further analysis.

### 2.5. Western Blot Analysis

Cells and tissues were lysed in RIPA buffer containing protease and phosphatase inhibitors (Beyotime, Shanghai, China). An amount of 15 μg of total protein per sample was separated by SDS-PAGE and transferred onto PVDF membranes (Millipore, Burlington, MA, USA). After blocking with 5% non-fat milk, membranes were incubated overnight at 4 °C with primary antibodies against NSUN4 (Proteintech, Wuhan, China; 29786-1-AP), C-MYC (Proteintech, Wuhan, China; 10828-1-AP), PI3K p85 (Cell Signaling Technology, Danvers, MA, USA; 4257T), phospho-PI3K p85 (Tyr458)/p55 (Tyr199) (Cell Signaling Technology, Danvers, MA, USA; 4228T), Akt (pan) (Cell Signaling Technology, Danvers, MA, USA; 4685T), phospho-Akt (Ser473) (Cell Signaling Technology, Danvers, MA, USA; 4060T), SLC7A11 (Proteintech, Wuhan, China; 26864-1-AP), GPX4 (Proteintech, Wuhan, China; 30388-1-AP), FTH1 (Proteintech, Wuhan, China; 11682-1-AP), Cyclin D1 (Proteintech, Wuhan, China; 26939-1-AP), CDK2 (Proteintech, Wuhan, China; 10122-1-AP), and GAPDH (Proteintech, Wuhan, China; 81640-5-RR). After washing, membranes were probed with HRP-conjugated secondary antibodies (ZSGB-BIO, Beijing, China; ZB-2301) at a dilution of 1:20000. Signals were visualized using enhanced chemiluminescence (ECL; NCM Biotech, Suzhou, China). Densitometric analysis was performed using ImageJ 1.54s software.

### 2.6. Tissue Microarray (TMA) and Immunohistochemistry (IHC)

A tissue microarray (TMA) containing 30 cervical cancer tissues and 30 adjacent non-cancerous tissues was utilized for this study. For the IHC experiments, the TMA sections were dewaxed, rehydrated, and subjected to antigen retrieval. Subsequently, the sections were incubated with an anti-NSUN4 primary antibody (Proteintech, Wuhan, China; 29786-1-AP) overnight at 4 °C, followed by incubation with the appropriate secondary antibody. Immunoreactivity was visualized using DAB, followed by hematoxylin counterstaining.

The stained TMA sections were quantitatively analyzed using the AI-based Aipathwell^®^ software (version 2.1.1, Servicebio, Wuhan, China). The software automatically identified tissue regions and evaluated staining intensity based on the Hue, Saturation, and Intensity (HSI) color model. Staining intensity was classified into four categories: negative (0), weak positive (1), moderate positive (2), and strong positive (3). The final protein expression of NSUN4 was represented by the Histochemistry score (H-score), ranging from 0 to 300, calculated as follows: H-score = (% of weak intensity cells × 1) + (% of moderate intensity cells × 2) + (% of strong intensity cells × 3).

### 2.7. Cell Proliferation and Colony Formation Assays

Cell proliferation was assessed using the CCK-8 kit (Dojindo, Mashiki, Kumamoto, Japan). Briefly, 2 × 10^3^ cells per well were seeded in 96-well plates. At indicated time points, 10 µL of CCK-8 reagent was added to each well, and absorbance at 450 nm was measured after 2 h incubation. For colony formation, 500 cells were plated in 6-well plates and cultured for 14 days. Colonies were fixed with 4% paraformaldehyde, stained with 0.1% crystal violet, and counted under a microscope.

### 2.8. Migration and Invasion Assays

Cell migration and invasion were evaluated using Transwell chambers (Corning, Corning, NY, USA). For invasion assays, the upper chambers were pre-coated with Matrigel (BD Biosciences, San Jose, CA, USA). Cells (5 × 10^4^) in serum-free medium were seeded into the upper chamber, and medium containing 10% FBS was added to the lower chamber as chemoattractant. After 24 h (migration) or 48 h (invasion), non-migrated cells were removed, and migrated cells were fixed, stained, and counted in five random fields per well under 200× magnification.

### 2.9. Generation and Treatment of Cervical Cancer Xenografts in Mice

Female BALB/c nude mice (6 weeks old) were randomly assigned to two groups (*n* = 6 per group). HeLa cells (5 × 10^6^) stably expressing sh-NSUN4 or sh-NC were subcutaneously injected into the right flank. Tumor volume was monitored every 3 days using the following formula: Volume = 0.5 × Length × Width^2^ (mm^3^). Mice were sacrificed 22 days post-injection, and tumors were harvested for weighing. The animal study protocol was reviewed and approved by the Animal Ethical and Welfare Committee (AEWC) of Beijing Keweite Animal Technology Co., Ltd. (Approval No. KWT-2025-10-12-01).

### 2.10. Inhibitor Treatment and Ferroptosis-Related Experiments

To assess the involvement of ferroptosis and the PI3K/AKT signaling pathway, cells were treated for 24 h with the apoptosis inhibitor Z-VAD-FMK (MedChemExpress, Monmouth Junction, NJ, USA), the necroptosis inhibitor Nec-1 (MedChemExpress, Monmouth Junction, NJ, USA), the ferroptosis inhibitors Ferrostatin-1 (MedChemExpress, Monmouth Junction, NJ, USA) and Liproxstatin-1 (MedChemExpress, Monmouth Junction, NJ, USA), or 3-Methyladenine (MedChemExpress, Monmouth Junction, NJ, USA), which was used in this study as an inhibitor of the PI3K pathway. Intracellular glutathione (GSH) levels were measured using a commercial assay kit (Beyotime, Shanghai, China) according to the manufacturer’s instructions. The expression levels of ferroptosis-related proteins, including SLC7A11, GPX4, and FTH1, were analyzed by Western blot as described above.

### 2.11. RNA Immunoprecipitation (RIP) and m^5^C-RIP Assays

RIP and m^5^C-RIP assays were performed using the Magna RIP RNA-Binding Protein Immunoprecipitation Kit (Millipore, Burlington, MA, USA). Briefly, cell lysates were incubated with magnetic beads conjugated with anti-NSUN4 (Proteintech, Wuhan, China) or anti-m^5^C antibodies overnight at 4 °C. After RNA purification, enrichment of C-MYC mRNA was detected by qRT-PCR. Normal IgG (Cell Signaling Technology, Danvers, MA, USA) served as the negative control.

### 2.12. RNA Stability and Dual-Luciferase Reporter Assays

For RNA stability analysis, transcription was blocked with actinomycin D (5 µg/mL), and cells were collected at 0, 2, 4, 6, and 8 h after treatment for RNA extraction. C-MYC mRNA half-life was determined by qRT-PCR. For luciferase reporter assays, wild-type or m^5^C-site-mutated fragments of the C-MYC 3′-UTR were cloned into the pGL4.10 vector (Promega, Madison, WI, USA). HEK-293T cells were co-transfected with the reporter construct and NSUN4 overexpression or control plasmids. After 48 h, luciferase activity was measured using the Dual-Luciferase Reporter Assay System (Promega, Madison, WI, USA).

### 2.13. Statistical Analysis

All experiments were repeated at least three times, and data are presented as the mean ± SD. Statistical analyses were performed using GraphPad Prism 9.0 and R software (version 4.2.2). Student’s *t*-test was used for comparisons between two groups, while one-way ANOVA was used for comparisons among multiple groups. Paired tissue comparisons were analyzed using paired Student’s *t*-test. Progression-free survival was analyzed using the Kaplan–Meier method and compared using the log-rank test. Tumor growth curves were analyzed using two-way ANOVA. A *p* value < 0.05 was considered statistically significant.

## 3. Results

### 3.1. NSUN4 Is Upregulated in Cervical Cancer and Correlated with Poor Prognosis in Patients with Cervical Cancer

**Figure 1:**
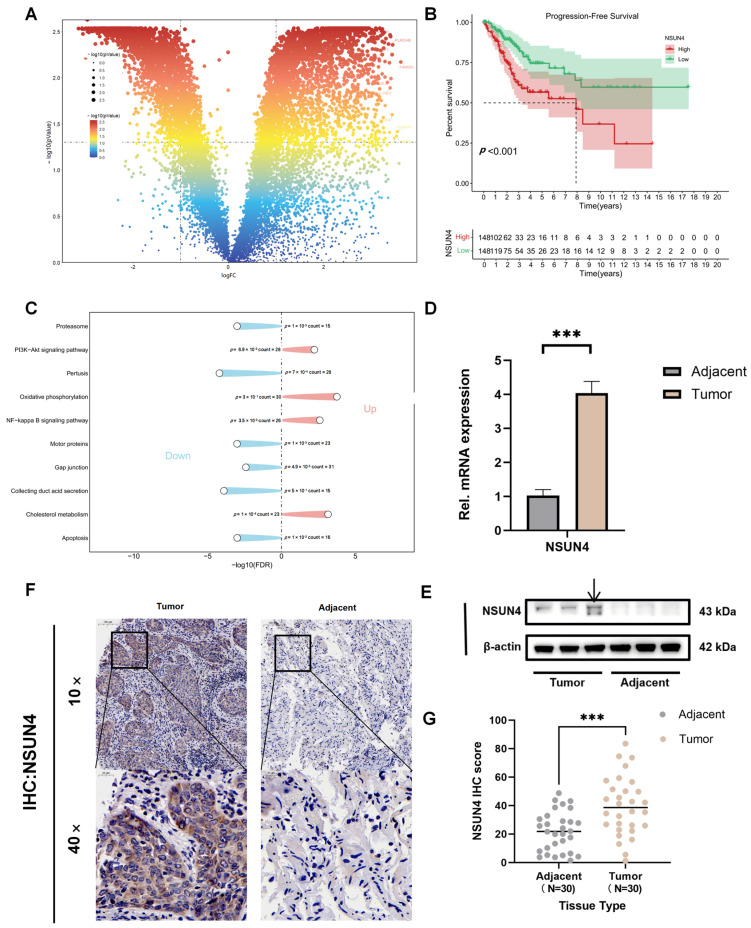
NSUN4 is upregulated in cervical cancer and associated with poor patient prognosis. (**A**) Differential expression analysis of TCGA RNA-sequencing data showing that NSUN4 is significantly upregulated in cervical cancer tissues compared with normal tissues. (**B**) Kaplan–Meier survival curves demonstrating that patients with high NSUN4 expression exhibit significantly worse progression-free survival than those with low expression (*p* < 0.05). (**C**) Gene Ontology (GO) and Kyoto Encyclopedia of Genes and Genomes (KEGG) enrichment analyses indicating that NSUN4-associated genes are mainly involved in apoptosis, PI3K–Akt signaling, and oxidative phosphorylation pathways. (**D**,**E**) Validation of NSUN4 expression in paired cervical cancer and adjacent normal tissues by qRT-PCR and Western blot, confirming elevated NSUN4 levels at both mRNA and protein levels in tumor samples (Supplementary Table S1). The arrow indicates the specific NSUN4 band. (**F**) Representative NSUN4 IHC staining images of cervical cancer and matched adjacent normal tissues from a tissue microarray (TMA). (**G**) Analysis of IHC scores from a cohort of 30 cervical cancer patients, showing the comparison of NSUN4 expression between cervical cancer and matched adjacent normal tissues. Statistical analysis was performed using a paired Student’s *t*-test (*** *p* < 0.001).

To identify key molecular drivers of cervical cancer, we analyzed the RNA-sequencing data and corresponding clinical information from the TCGA-CESC cohort. Differential expression analysis between tumor and normal tissues identified a set of significantly dysregulated genes. Among these significantly altered genes, NSUN4 was selected for further investigation because it showed marked upregulation in tumor samples in the volcano plot (Figure 1A). Specifically, NSUN4 showed a fold change of 9.7946 and a *p* value of 0.0251 in the TCGA dataset.

To determine the clinical relevance of NSUN4 expression, we performed Kaplan–Meier survival analysis, which revealed that patients with high NSUN4 expression had significantly poorer progression-free survival compared with those exhibiting low expression levels (*p* < 0.05) (Figure 1B). Next, we performed GO and KEGG enrichment analyses. The enriched terms were primarily associated with apoptosis, PI3K-Akt signaling pathway and oxidative phosphorylation, suggesting that these pathways may play a role in cervical cancer pathogenesis (Figure 1C). Finally, to validate the bioinformatic findings, we analyzed three paired cervical cancer and adjacent normal tissues. Both qRT-PCR and Western blot assays confirmed that NSUN4 expression was significantly elevated at the mRNA and protein levels in tumor tissues relative to adjacent benign tissues (Figure 1D,E). The direction of NSUN4 upregulation observed in our RT-qPCR results was consistent with that identified in the TCGA-CESC dataset. We performed IHC in a tissue microarray (TMA) of patient samples. Through quantitative IHC analysis, we confirmed that NSUN4 protein levels were much higher in cancerous tissues compared to adjacent benign tissues (Figure 1F,G).

Together, these results identify NSUN4 as a clinically relevant gene that is markedly upregulated in cervical cancer and may contribute to its malignant progression.

### 3.2. NSUN4 Enhances Proliferation, Migration, and Invasion of Cervical Cancer Cells

**Figure 2:**
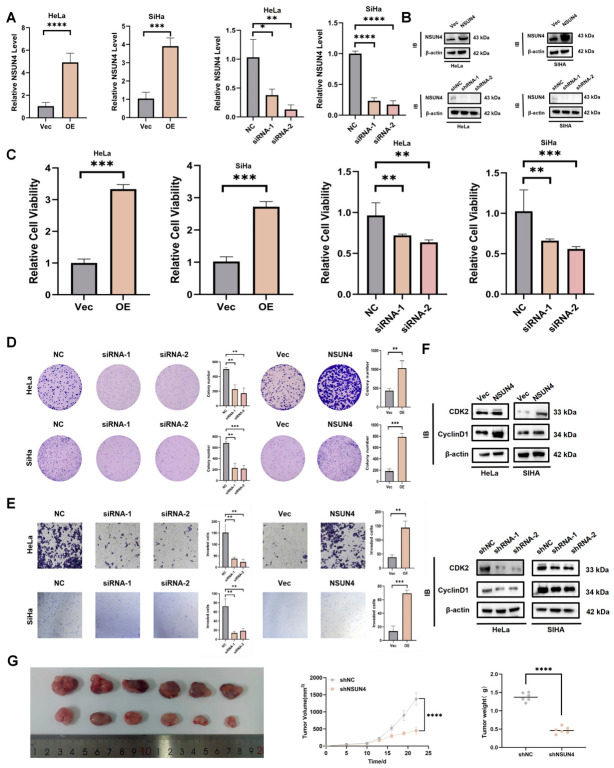
NSUN4 promotes proliferation, migration, and invasion of cervical cancer cells. (**A**,**B**) Validation of NSUN4 overexpression and knockdown efficiency in cervical cancer cell lines using qRT-PCR and Western blot analysis. (**C**,**D**) CCK-8 and colony formation assays showing that NSUN4 overexpression significantly enhances whereas NSUN4 silencing suppresses cervical cancer cell proliferation. (**E**) Transwell migration and invasion assays demonstrating that NSUN4 upregulation promotes while NSUN4 knockdown inhibits the migratory and invasive capacities of cervical cancer cells. (**F**) Western blot analysis of cell cycle proteins indicating that NSUN4 overexpression increases cell cycle progression, whereas NSUN4 depletion induces cell cycle arrest. (**G**) Representative images of subcutaneous xenograft tumors harvested from nude mice (**left**), and statistical analysis of tumor volume (**middle**) and tumor weight (**right**) in sh-NC and sh-NSUN4 groups (*n* = 6 per group). Data are presented as mean ± SD, * *p* < 0.05, ** *p* < 0.01, *** *p* < 0.001, **** *p* < 0.0001.

To determine the tumorigenic function of NSUN4 in cervical cancer, we first validated the efficiency of NSUN4 overexpression and knockdown in cervical cancer cells by qPCR and Western blot (Figure 2A,B). Functional assays demonstrated that NSUN4 overexpression significantly promoted cell proliferation, as evidenced by CCK-8 and colony formation assays, whereas NSUN4 silencing markedly suppressed proliferation (Figure 2C,D). Transwell assays revealed that NSUN4 upregulation enhanced cell invasion, while NSUN4 knockdown inhibited these malignant behaviors (Figure 2E). Mechanistically, Western blot analysis showed that NSUN4 overexpression increased the levels of cell cycle–related proteins. Flow cytometric analysis further confirmed that NSUN4 depletion caused G0/G1 phase arrest (Supplementary Figure S1). To further validate these findings in vivo, a nude mouse xenograft model was established. The results showed that NSUN4 knockdown significantly inhibited tumor growth (Figure 2G). Both tumor volume and weight were markedly reduced in the sh-NSUN4 group compared with the control group (*p* < 0.001).

Together, these findings indicate that NSUN4 exerts oncogenic effects in cervical cancer by promoting proliferation, migration, and invasion.

### 3.3. NSUN4 Promotes Cervical Cancer Cell Growth by Suppressing Ferroptosis

**Figure 3:**
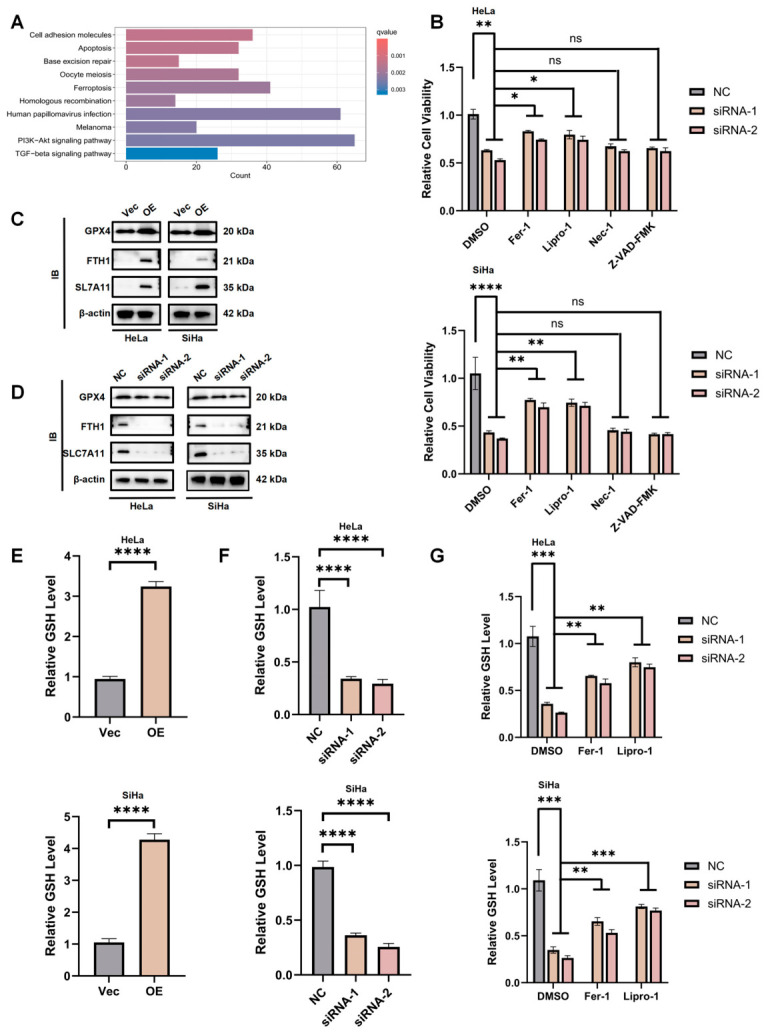
NSUN4 promotes cervical cancer cell proliferation by suppressing ferroptosis. (**A**) KEGG pathway enrichment analysis of transcriptome sequencing data from NSUN4-overexpressing and control cells revealing a significant association with ferroptosis pathways. (**B**) Cell viability assays showing that ferroptosis inhibitors (Fer-1 and Lipro-1), but not apoptosis or necroptosis inhibitors (Z-VAD-FMK or Nec-1), rescue the proliferation defects induced by NSUN4 knockdown. (**C**,**D**) Western blot analysis demonstrating that NSUN4 depletion downregulates, whereas NSUN4 overexpression upregulates, ferroptosis-associated proteins SLC7A11, FTH1, and GPX4. (**E**–**G**) Quantification of intracellular GSH levels showing that NSUN4 overexpression increases, while NSUN4 knockdown decreases, GSH content; ferroptosis inhibition with Fer-1 or Lipro-1 restores GSH levels in NSUN4-deficient cells. Data are presented as mean ± SD. ns, not significant; * *p* < 0.05, ** *p* < 0.01, *** *p* < 0.001, **** *p* < 0.0001.

To explore the mechanisms by which NSUN4 regulates cervical cancer cell phenotypes, we performed transcriptome sequencing of NSUN4-overexpressing and control cells. KEGG indicated a significant association with ferroptosis pathways (Figure 3A). To verify this finding, we treated NSUN4-deficient cells with inhibitors of apoptosis (Z-VAD-FMK), necroptosis (Nec-1), or ferroptosis (Fer-1 and Lipro-1). Only Fer-1 and Lipro-1 rescued the proliferation defects induced by NSUN4 knockdown, suggesting that NSUN4 regulates cell growth through ferroptosis inhibition (Figure 3B). Furthermore, Western blot analysis demonstrated that NSUN4 depletion downregulated ferroptosis-associated proteins (SLC7A11, FTH1, and GPX4), while NSUN4 overexpression upregulated their expression (Figure 3C,D). Consistently, NSUN4 overexpression elevated intracellular GSH levels, whereas NSUN4 knockdown reduced GSH content, which was restored by Fer-1 or Lipro-1 (Figure 3E–G).

Together, these results indicate that NSUN4 promotes cervical cancer cell survival and proliferation by suppressing ferroptosis.

### 3.4. NSUN4 Suppresses Ferroptosis Through Activation of the PI3K/Akt Pathway

**Figure 4:**
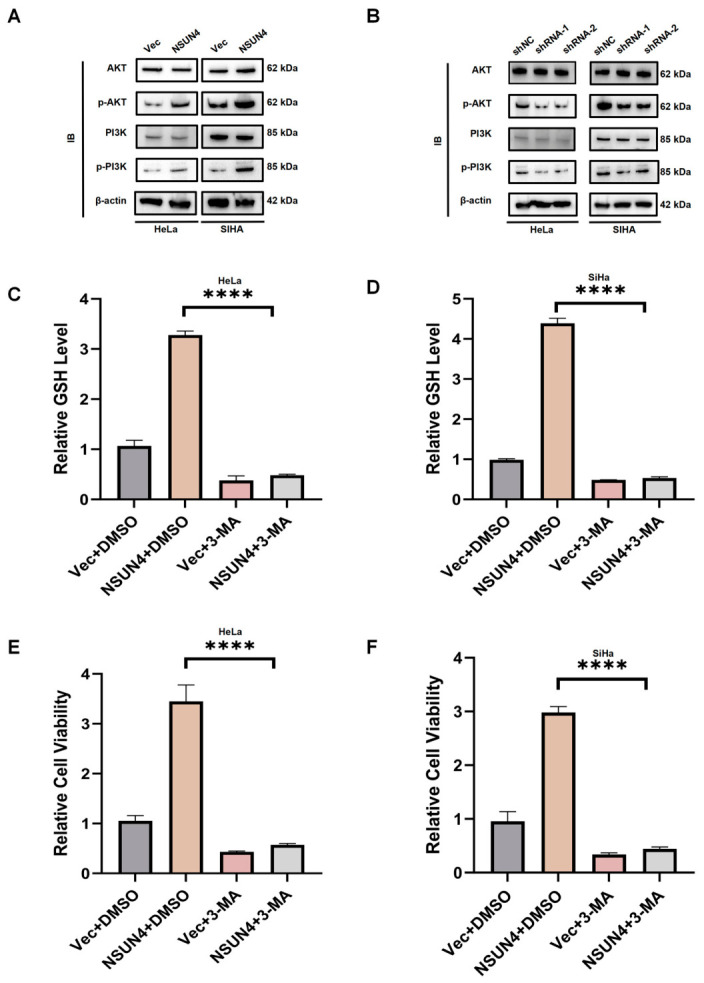
NSUN4 suppresses ferroptosis by activating the PI3K/Akt signaling pathway. (**A**,**B**) Western blot analysis showing that NSUN4 overexpression enhances whereas NSUN4 knockdown reduces phosphorylation levels of PI3K and Akt, indicating activation of the PI3K/Akt pathway. (**C**,**D**) Measurement of intracellular GSH levels demonstrating that treatment with a PI3K/Akt inhibitor decreases GSH content in NSUN4-overexpressing cervical cancer cells. (**E**,**F**) CCK-8 assays revealing that pharmacologic inhibition of PI3K/Akt signaling attenuates the NSUN4-induced proliferative advantage in cervical cancer cells. Data are presented as mean ± SD, **** *p* < 0.0001.

Based on enrichment analyses and literature review, we hypothesized that the PI3K/Akt pathway mediates NSUN4-regulated ferroptosis. Western blot confirmed that NSUN4 overexpression enhanced PI3K/Akt pathway activation, whereas NSUN4 knockdown reduced its activity (Figure 4A,B).

To determine whether PI3K/Akt signaling is required for NSUN4-mediated ferroptosis suppression, we treated NSUN4-overexpressing cells with a PI3K/Akt inhibitor. GSH measurements showed that PI3K/Akt inhibition reduced GSH levels in NSUN4-overexpressing cells (Figure 4C,D). Moreover, CCK-8 assays revealed that blocking PI3K/Akt signaling attenuated the proliferative advantage conferred by NSUN4 overexpression (Figure 4E,F).

These findings indicate that NSUN4 suppresses ferroptosis and promotes cervical cancer cell proliferation by activating the PI3K/Akt pathway.

### 3.5. NSUN4 Stabilizes C-MYC mRNA via m^5^C Modification to Activate the PI3K/Akt Pathway

**Figure 5:**
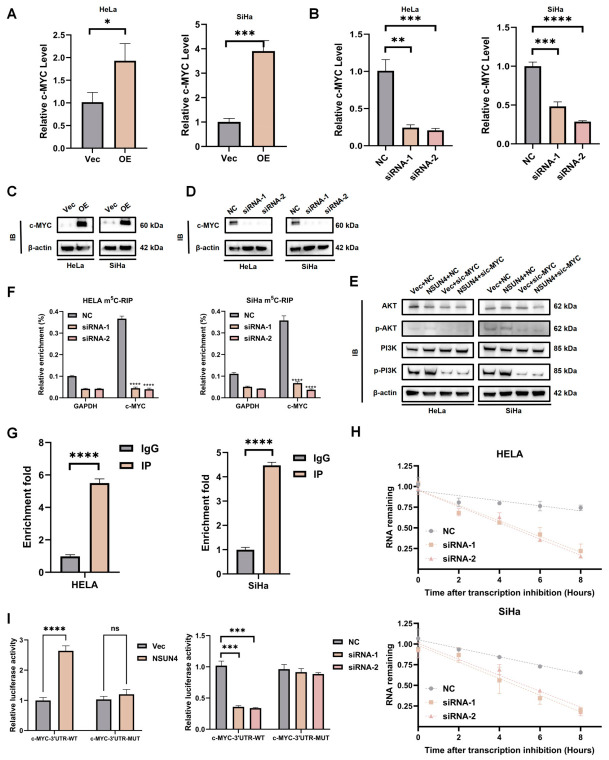
NSUN4 stabilizes C-MYC mRNA via m^5^C modification to activate the PI3K/Akt pathway. (**A**–**D**) qRT-PCR and Western blot analyses showing that NSUN4 overexpression increases while NSUN4 knockdown decreases C-MYC mRNA and protein expression levels in cervical cancer cells. (**E**) Western blot analysis demonstrating that C-MYC silencing abolishes PI3K/Akt pathway activation induced by NSUN4 overexpression, indicating that C-MYC mediates NSUN4-driven PI3K/Akt signaling. (**F**) m^5^C-RIP assays showing reduced C-MYC mRNA methylation upon NSUN4 knockdown. (**G**) RIP–qPCR confirming that NSUN4 directly binds to C-MYC transcripts. (**H**) RNA stability assays revealing that the half-life of C-MYC mRNA is shortened in NSUN4-deficient cells. (**I**) Dual-luciferase reporter assays showing that NSUN4 enhances luciferase activity through the wild-type C-MYC 3′UTR, whereas mutation of the m^5^C sites abolishes this effect. Data are presented as mean ± SD, ns, not significant; * *p* < 0.05, ** *p* < 0.01, *** *p* < 0.001, **** *p* < 0.0001.

To further elucidate how NSUN4 regulates the PI3K/Akt pathway, we analyzed the differentially expressed genes and identified C-MYC as a potential downstream effector. qPCR and Western blot assays showed that NSUN4 overexpression increased C-MYC expression, whereas NSUN4 knockdown reduced its expression (Figure 5A–D). Importantly, silencing C-MYC abrogated PI3K/Akt pathway activation induced by NSUN4 overexpression, indicating that C-MYC mediates NSUN4-driven PI3K/Akt pathway (Figure 5E). Mechanistically, m^5^C-RIP demonstrated that C-MYC mRNA methylation was reduced upon NSUN4 knockdown (Figure 5F). RIP-qPCR further confirmed that NSUN4 directly binds to C-MYC transcripts (Figure 5G). RNA stability assays revealed that C-MYC mRNA half-life was shortened in NSUN4-deficient cells (Figure 5H). Finally, dual-luciferase reporter assays showed that NSUN4 enhanced luciferase activity through the C-MYC 3′UTR, whereas mutation of the m^5^C sites abolished this effect (Figure 5I).

Collectively, these findings indicate that NSUN4 directly binds to and stabilizes C-MYC mRNA via m^5^C modification, thereby promoting PI3K/Akt pathway activation and cervical cancer progression.

## 4. Discussion

In this study, we identified NSUN4 as a novel oncogenic regulator that promotes cervical cancer progression by suppressing ferroptosis through the C-MYC/PI3K/Akt signaling axis. Our results demonstrate that NSUN4 is significantly upregulated in cervical cancer tissues, correlates with unfavorable prognosis, and enhances tumor cell proliferation, migration, and invasion. Mechanistically, NSUN4 stabilizes C-MYC mRNA via m^5^C methylation, thereby activating the PI3K/Akt pathway and increasing antioxidant capacity, which in turn inhibits ferroptosis and supports malignant growth. These findings provide new insights into the interplay between RNA methylation, oncogenic signaling, and regulated cell death in cervical cancer.

Ferroptosis represents a distinct form of iron-dependent cell death characterized by lipid peroxidation and disruption of redox homeostasis [18]. Mounting evidence suggests that evasion of ferroptosis is a critical feature of tumor aggressiveness and therapeutic resistance [21]. Our study extends this concept by identifying NSUN4 as a ferroptosis suppressor in cervical cancer. NSUN4 overexpression elevated intracellular GSH levels and increased expression of ferroptosis defense proteins (GPX4, SLC7A11, and FTH1), while its depletion led to ferroptotic vulnerability that was rescued by classical ferroptosis inhibitors. These observations indicate that NSUN4 protects tumor cells from oxidative stress-induced death, conferring a survival advantage under metabolic stress.

The mechanistic link between NSUN4 and ferroptosis appears to be mediated by PI3K/Akt signaling, a well-known pathway involved in cell survival and redox regulation [22]. Activation of PI3K/Akt has been reported to inhibit ferroptosis by maintaining GSH biosynthesis and reducing lipid peroxidation [23,24]. Consistent with these reports, our data show that NSUN4 overexpression enhances PI3K/Akt activation, whereas pharmacologic blockade of PI3K/Akt signaling abolishes the anti-ferroptotic and pro-proliferative effects of NSUN4. This suggests that PI3K/Akt serves as an essential downstream effector of NSUN4, linking RNA methylation to metabolic adaptation and cell fate control.

Importantly, we uncovered C-MYC as a direct downstream target of NSUN4. C-MYC is a master transcriptional regulator of cellular growth, metabolism, and survival, and its dysregulation is a hallmark of numerous cancers [25]. Our RIP and m^5^C-RIP assays revealed that NSUN4 directly binds to C-MYC transcripts and modifies them via m^5^C methylation, leading to enhanced mRNA stability. This modification prolongs the half-life of C-MYC mRNA and sustains its expression, which subsequently activates PI3K/Akt signaling and suppresses ferroptosis. This mechanism establishes a novel epitranscriptomic–oncogenic axis wherein NSUN4-mediated m^5^C methylation of C-MYC reinforces a feedback loop promoting tumor survival. Similar regulatory paradigms have been observed in other m^5^C writers, such as NSUN2, which stabilize oncogenic transcripts to promote tumor progression [26,27,28]. Our findings extend this concept to NSUN4, highlighting its pivotal role in linking RNA methylation to ferroptosis resistance.

From a translational standpoint, the identification of the NSUN4–C-MYC–PI3K/Akt axis opens new avenues for therapeutic intervention. Targeting ferroptosis resistance has recently emerged as a promising strategy in cancer therapy [19], and our study suggests that inhibiting NSUN4 or disrupting its downstream signaling could restore ferroptotic sensitivity in cervical cancer cells. Furthermore, because NSUN4 expression correlates with poor prognosis, it may serve as a prognostic biomarker or predictive indicator of ferroptosis-targeted therapeutic response. Combining NSUN4 inhibition with ferroptosis inducers or PI3K/Akt pathway inhibitors may therefore provide synergistic benefits in overcoming resistance to conventional treatments.

Nevertheless, several limitations of this study should be acknowledged. First, while our in vitro data demonstrate that NSUN4 regulates ferroptosis and tumor growth, in vivo validation using xenograft or orthotopic models is required to confirm its role in tumor progression and treatment response. Second, the precise structural basis of NSUN4 recognition and methylation of C-MYC transcripts remains to be elucidated. Future studies integrating CLIP-seq and m^5^C-seq analyses, methodologies that have successfully mapped the target networks of other epitranscriptomic regulators [29], could provide genome-wide mapping of NSUN4 binding sites and help identify additional downstream targets. Lastly, given that NSUN4 is also localized in mitochondria, it would be of interest to explore whether its mitochondrial functions contribute to metabolic reprogramming and ferroptosis regulation in cancer cells, as mitochondrial metabolism is increasingly recognized as a crucial driver of ferroptotic cell death [30].

In summary, our study reveals that NSUN4 acts as an m^5^C methyltransferase that stabilizes C-MYC mRNA, activates PI3K/Akt signaling, and suppresses ferroptosis, thereby promoting cervical cancer progression. These findings define a previously unrecognized NSUN4–C-MYC–PI3K/Akt–ferroptosis signaling axis, advancing our understanding of RNA methylation-mediated ferroptosis resistance and offering new therapeutic strategies for cervical cancer.

## 5. Conclusions

In conclusion, this study identifies NSUN4 as a crucial oncogenic driver in cervical cancer that promotes tumor progression by stabilizing C-MYC mRNA through m^5^C methylation, thereby activating the PI3K/Akt signaling pathway and conferring resistance to ferroptosis. By linking RNA methylation to oxidative stress adaptation, NSUN4 establishes a novel NSUN4–C-MYC–PI3K/Akt axis that integrates epitranscriptomic regulation with cell survival mechanisms. These findings not only elucidate a previously unrecognized layer of ferroptosis control in cervical cancer but also highlight NSUN4 as a promising biomarker and therapeutic target for ferroptosis-based cancer treatment.

## Data Availability

All data utilized in this research are publicly accessible. RNA-sequencing datasets were retrieved from The Cancer Genome Atlas (TCGA) database (https://portal.gdc.cancer.gov/, accessed on 1 January 2025). Additional data supporting the conclusions of this work can be obtained from the corresponding author upon reasonable request.

## Supplementary Materials

The following supporting information can be downloaded at: https://www.mdpi.com/article/10.3390/cancers18091392/s1, Figure S1: Original Western blot images corresponding to the main figures, including uncropped blot bands, molecular weight markers, and indication of the target protein bands; Table S1: Raw PCR data.
