# Supplementary material for: NSUN4 Suppresses Ferroptosis Through m5C-Dependent Stabilization of C-MYC and Activation of the PI3K/Akt Signaling Pathway in Cervical Cancer
Source: Cancers (Basel). 2026 Apr 28;18(9):1392. doi: 10.3390/cancers18091392 (PMC13162798; doi:10.3390/cancers18091392)

# Full unedited western blots

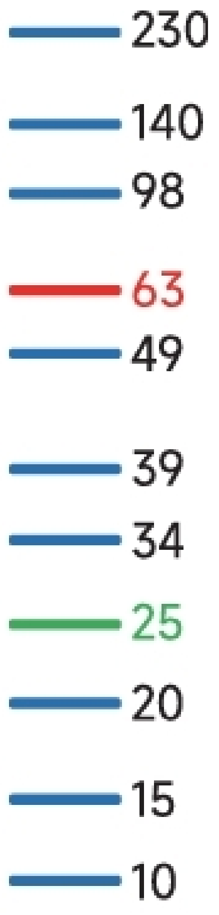


**The present study employed the marker as illustrated in the left panel**

# Full unedited western blots for Fig 2B

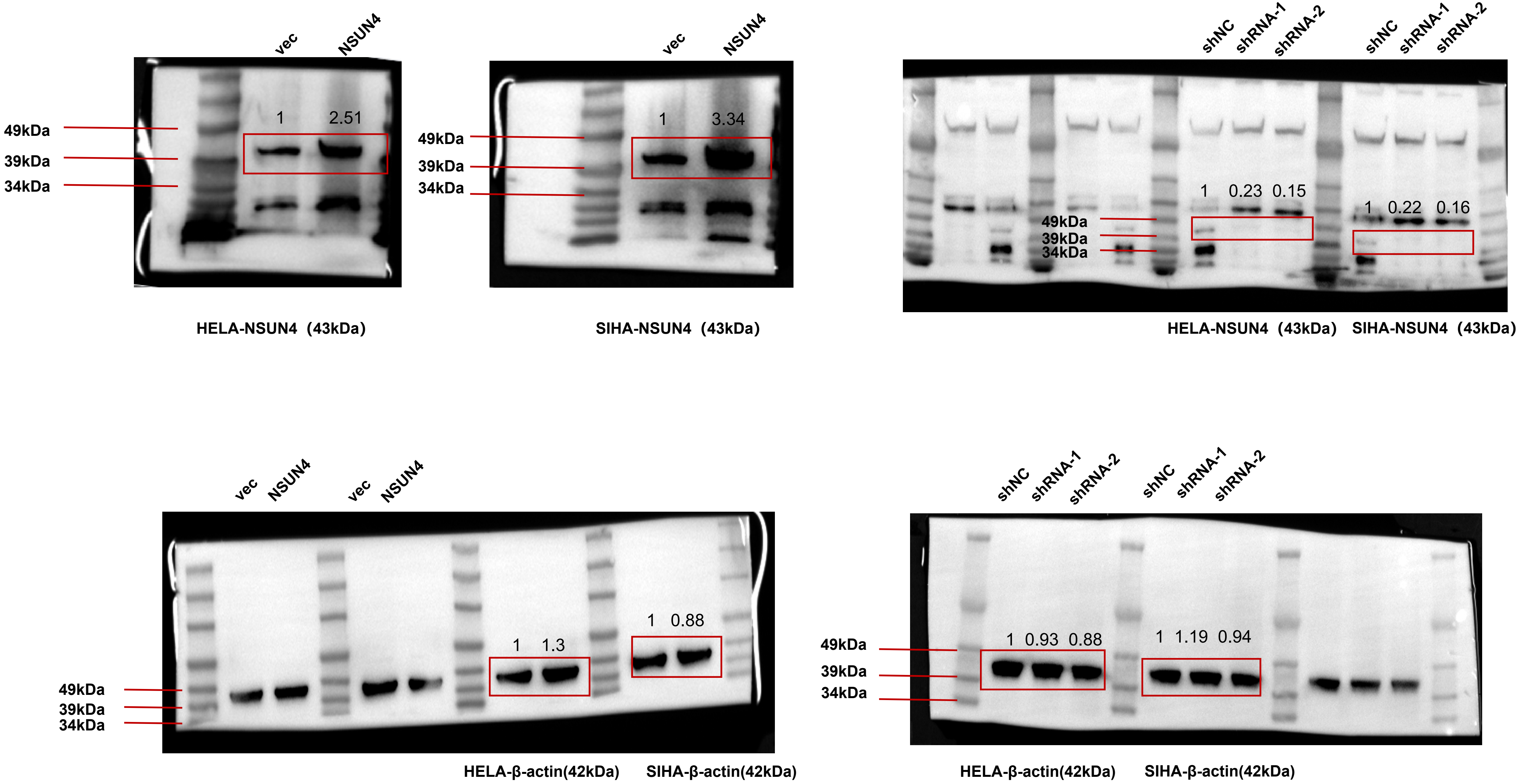

# Full unedited western blots for Fig 2F

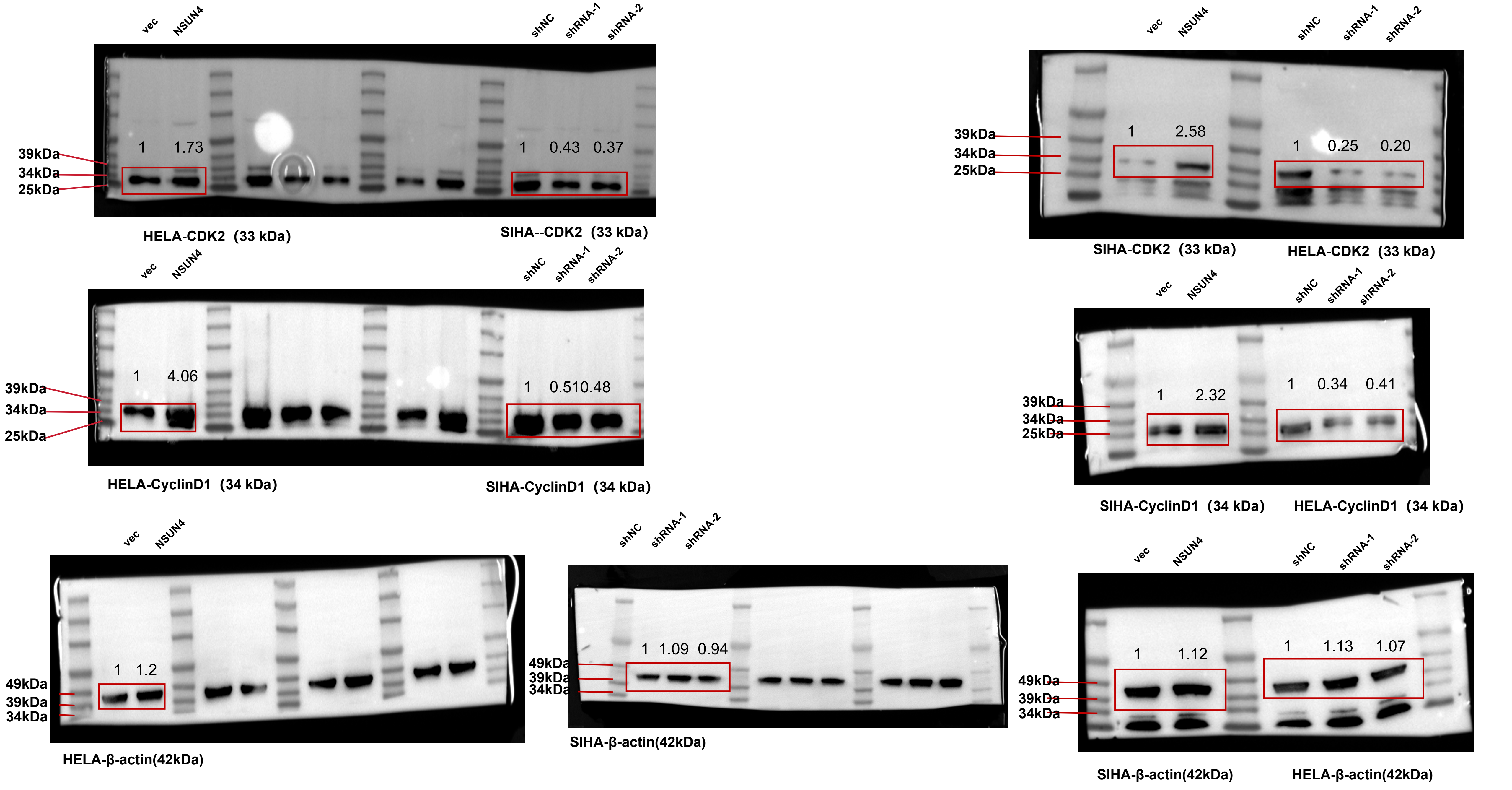

# Full unedited western blots for Fig 1D

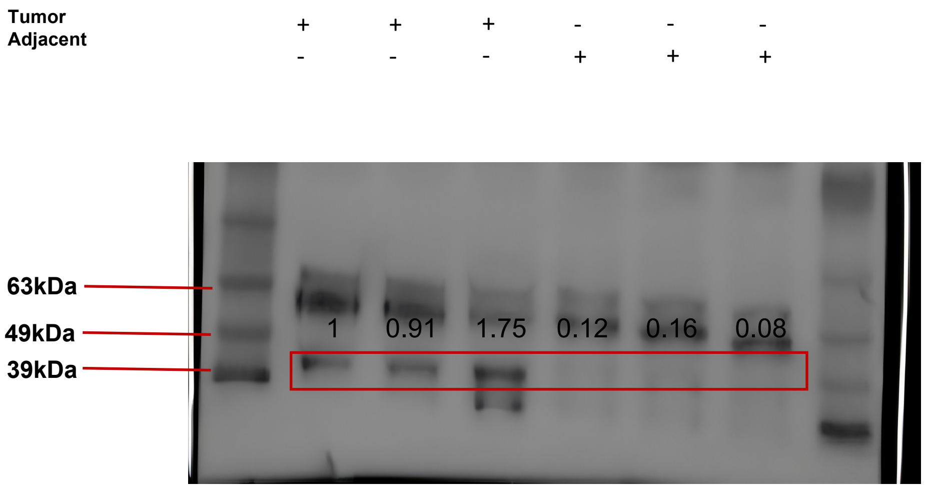


NSUN4 (43kDa)

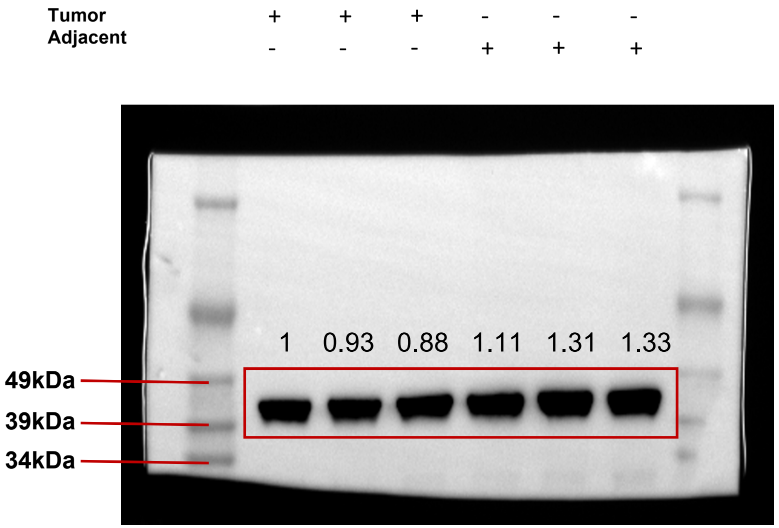


β-actin(42kDa)

# Full unedited western blots for Fig 3C

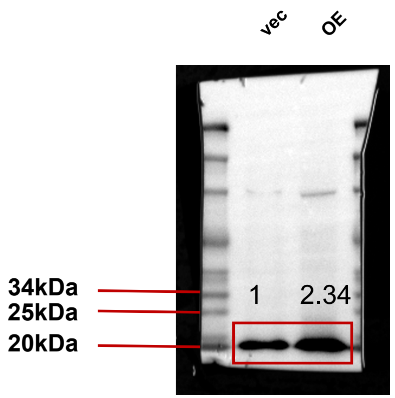


HELA-GPX4 (20 kDa)

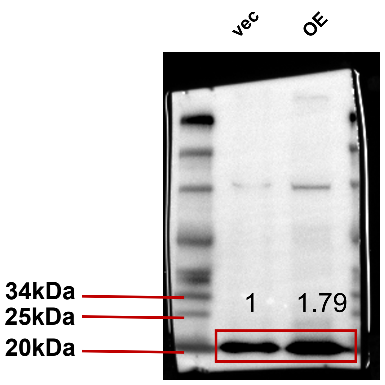


SIHA-GPX4 (20 kDa)

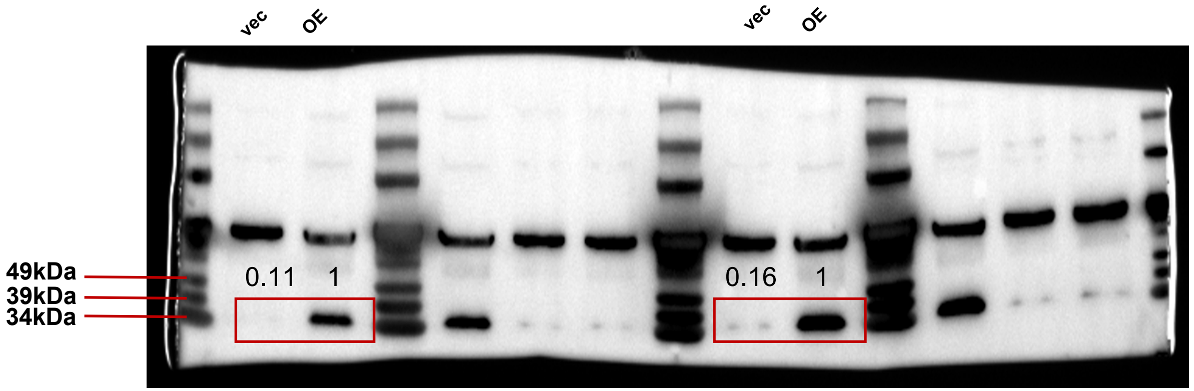


HELA-SL7A11(35kDa) SIHA-SL7A11(35kDa)

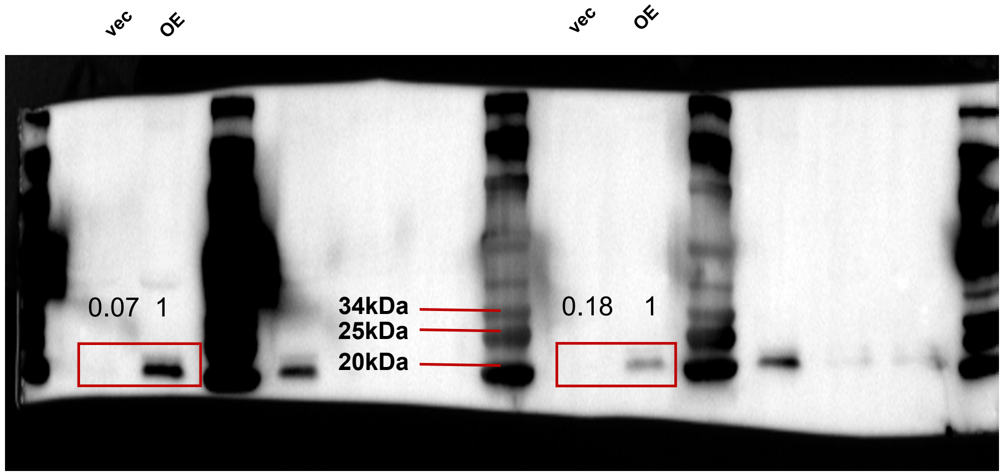


HELA-FTH1 (21 kDa) SIHA-FTH1 (21 kDa)

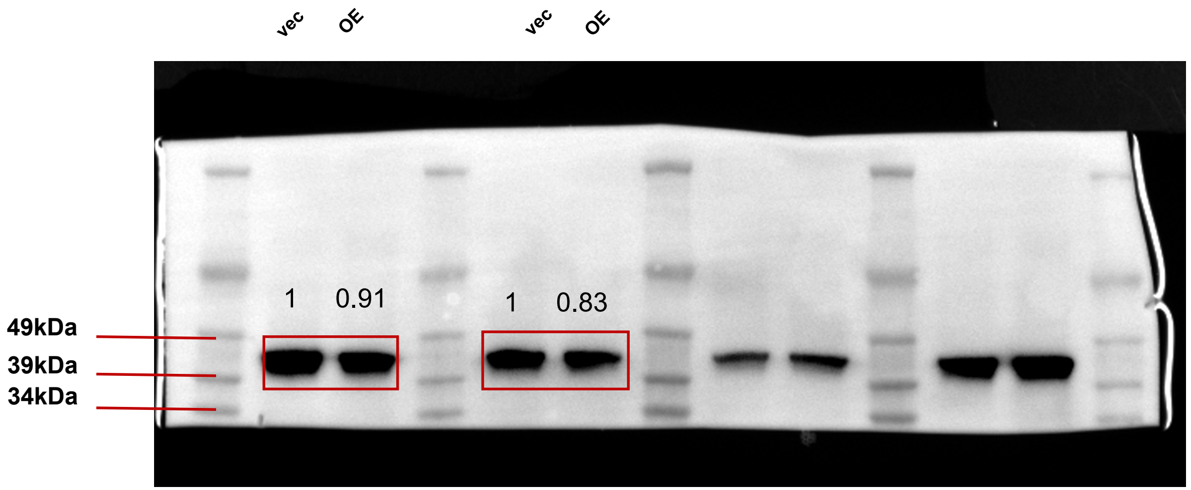


HELA-β-actin(42kDa) SIHA-β-actin(42kDa)

# Full unedited western blots for Fig 3D

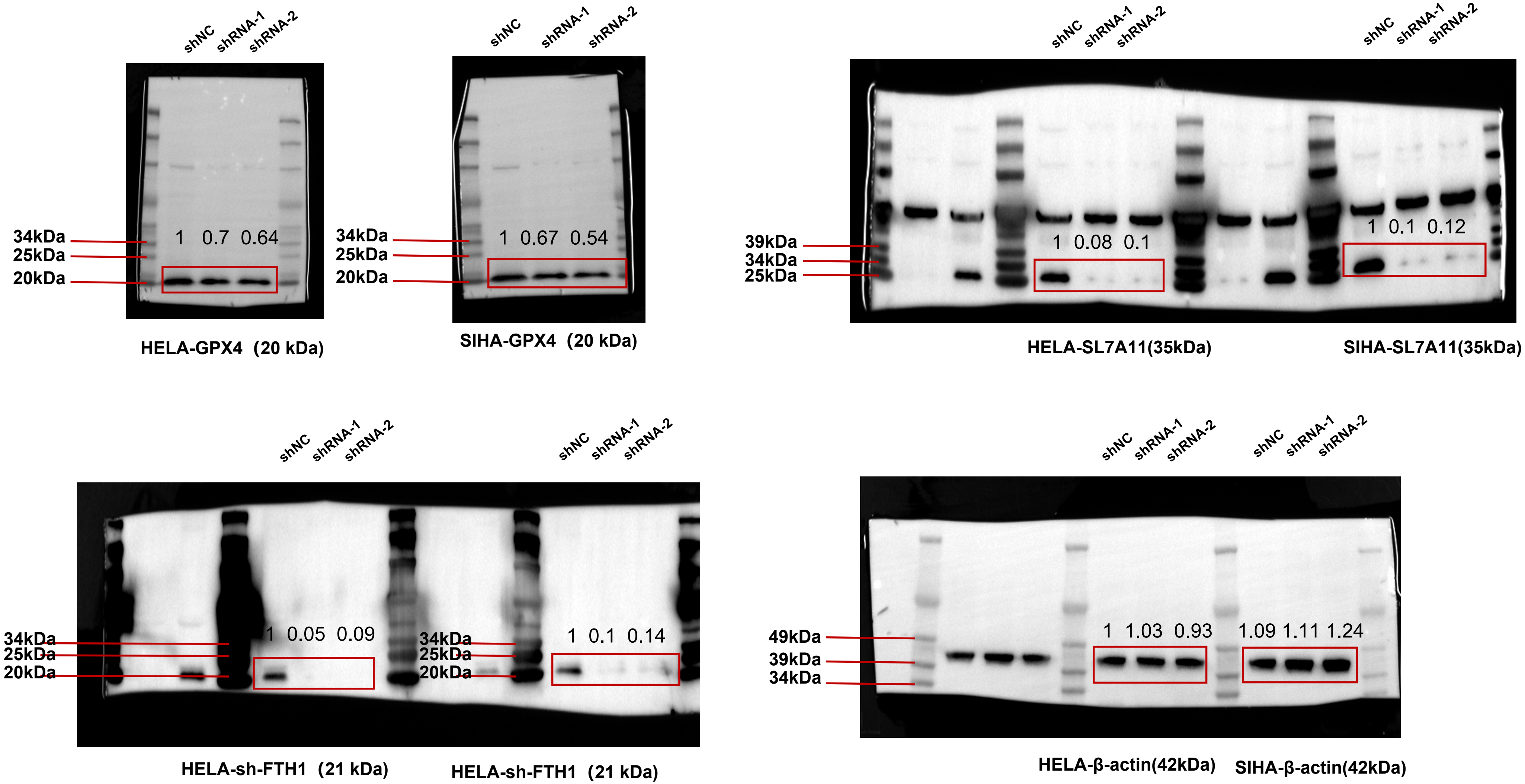

# Full unedited western blots for Fig 4A

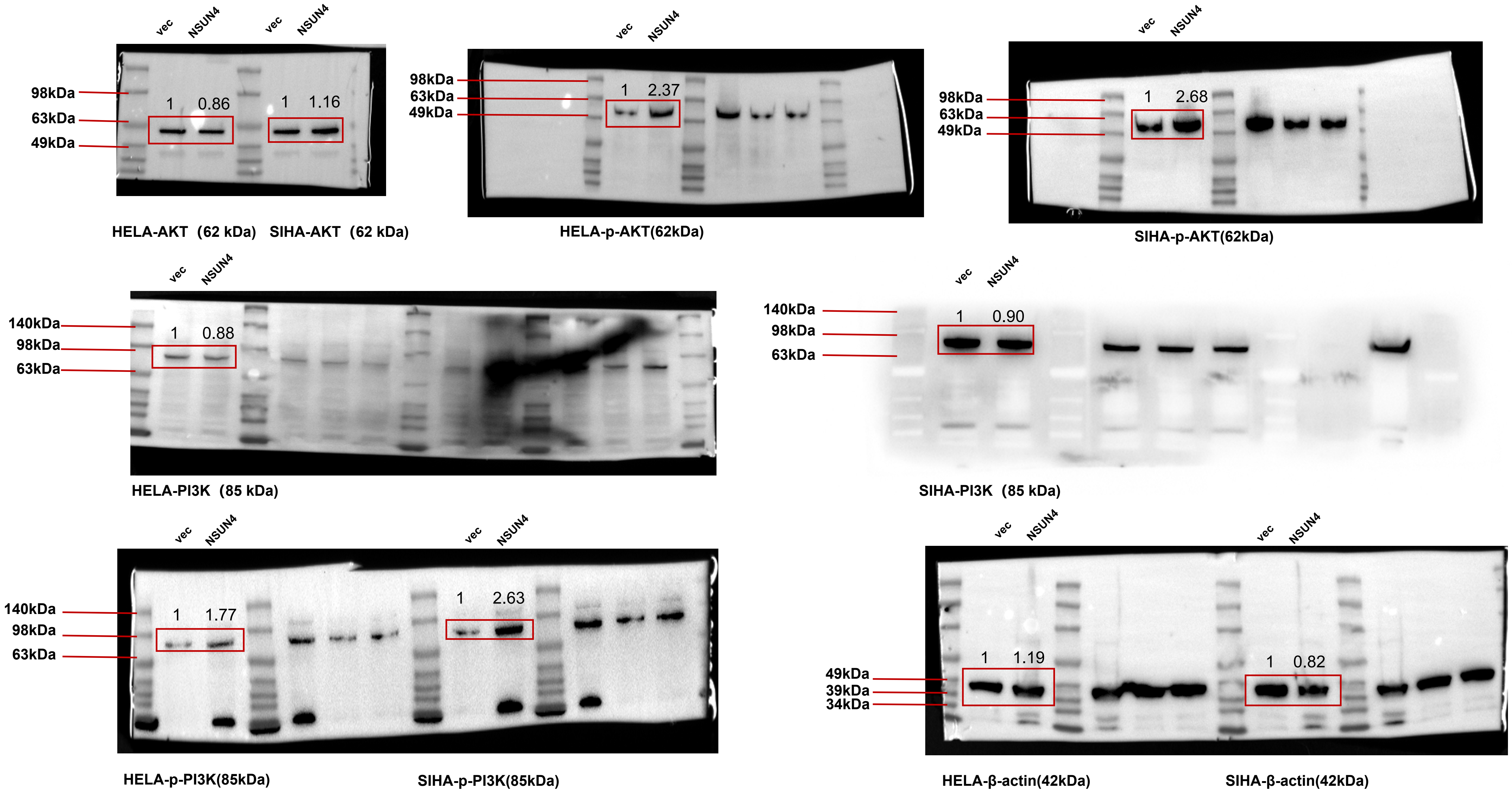

# Full unedited western blots for Fig 4B

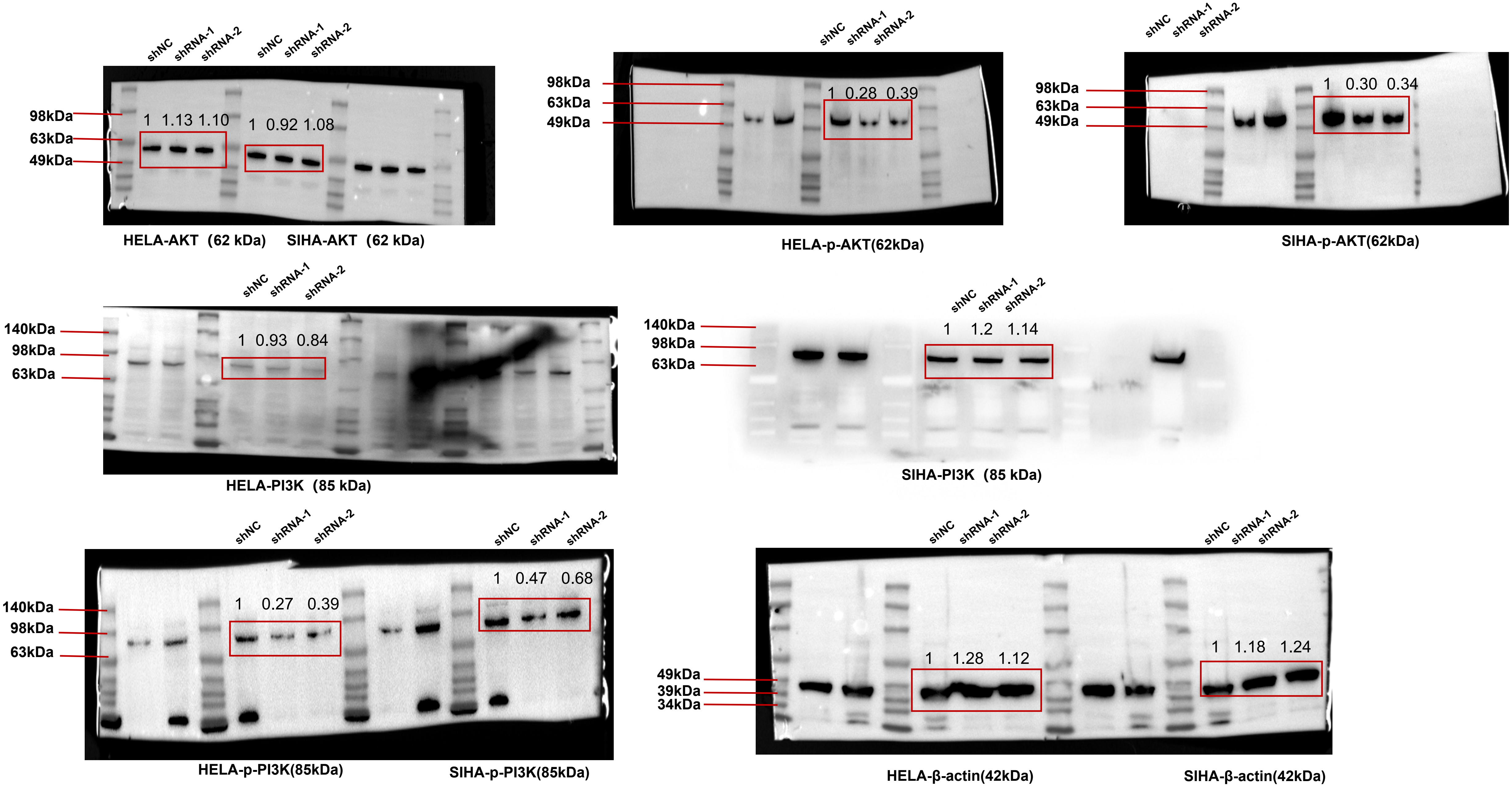

# Full unedited western blots for Fig 5C，D

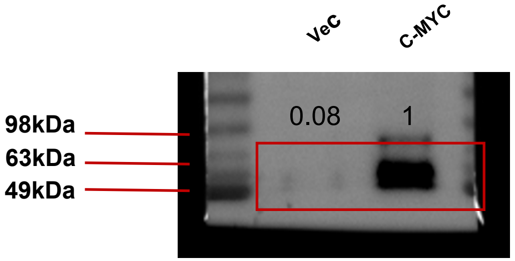


SIHA-C-MYC(60kDa)

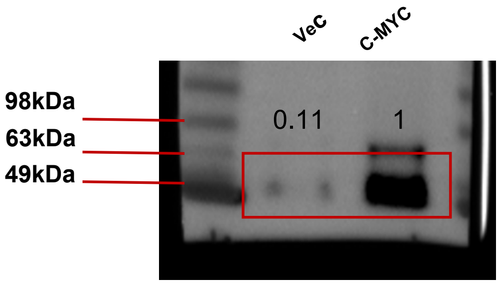


HELA-C-MYC(60kDa)

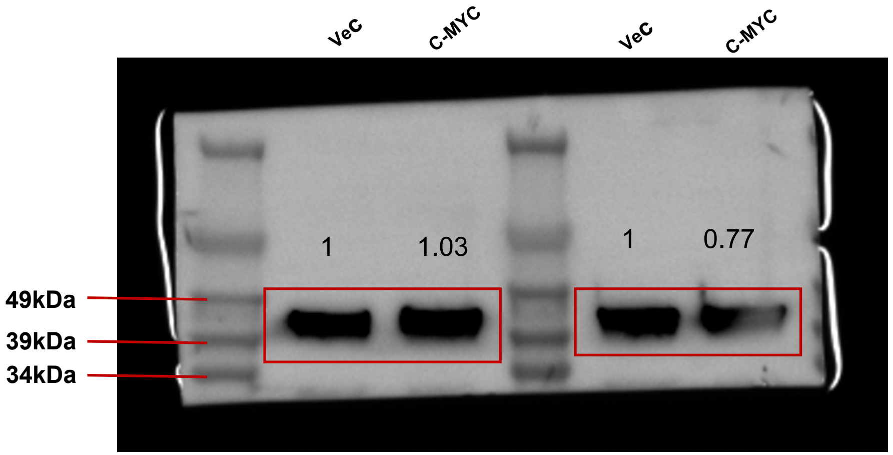


SIHA-β-actin(42kDa) HELA-β-actin(42kDa)

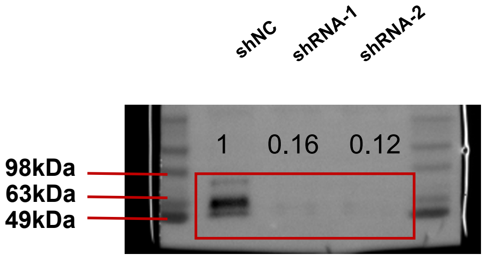


HELA-C-MYC(60kDa)

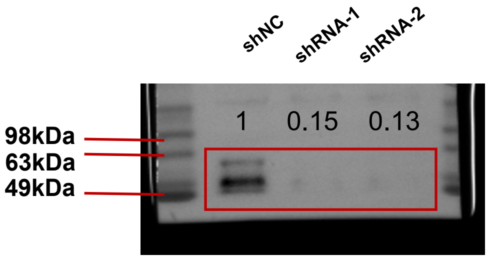


SIHA-C-MYC(60kDa)

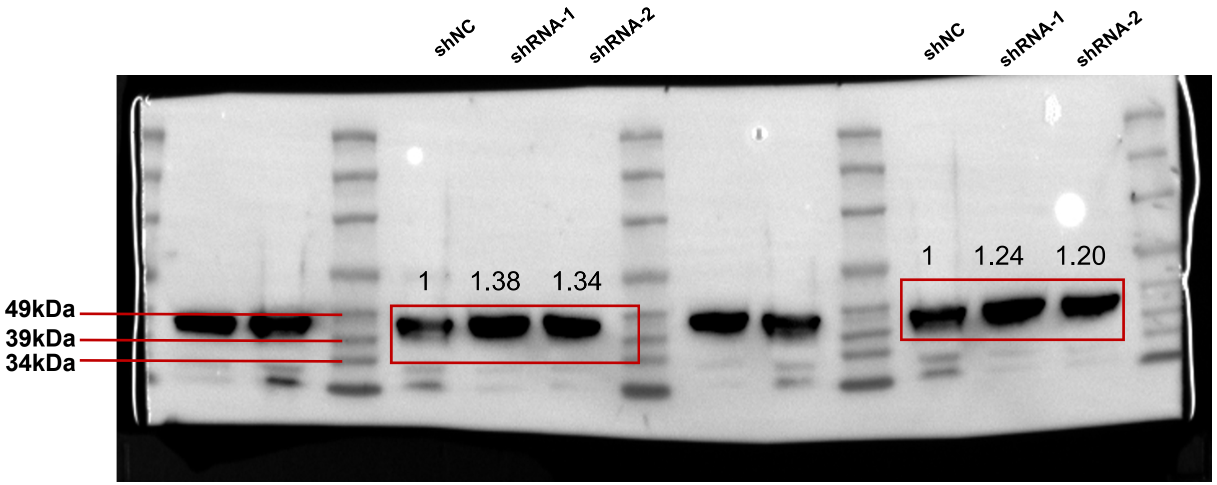


HELA-β-actin(42kDa) SIHA-β-actin(42kDa)

# Full unedited western blots for Fig 5E

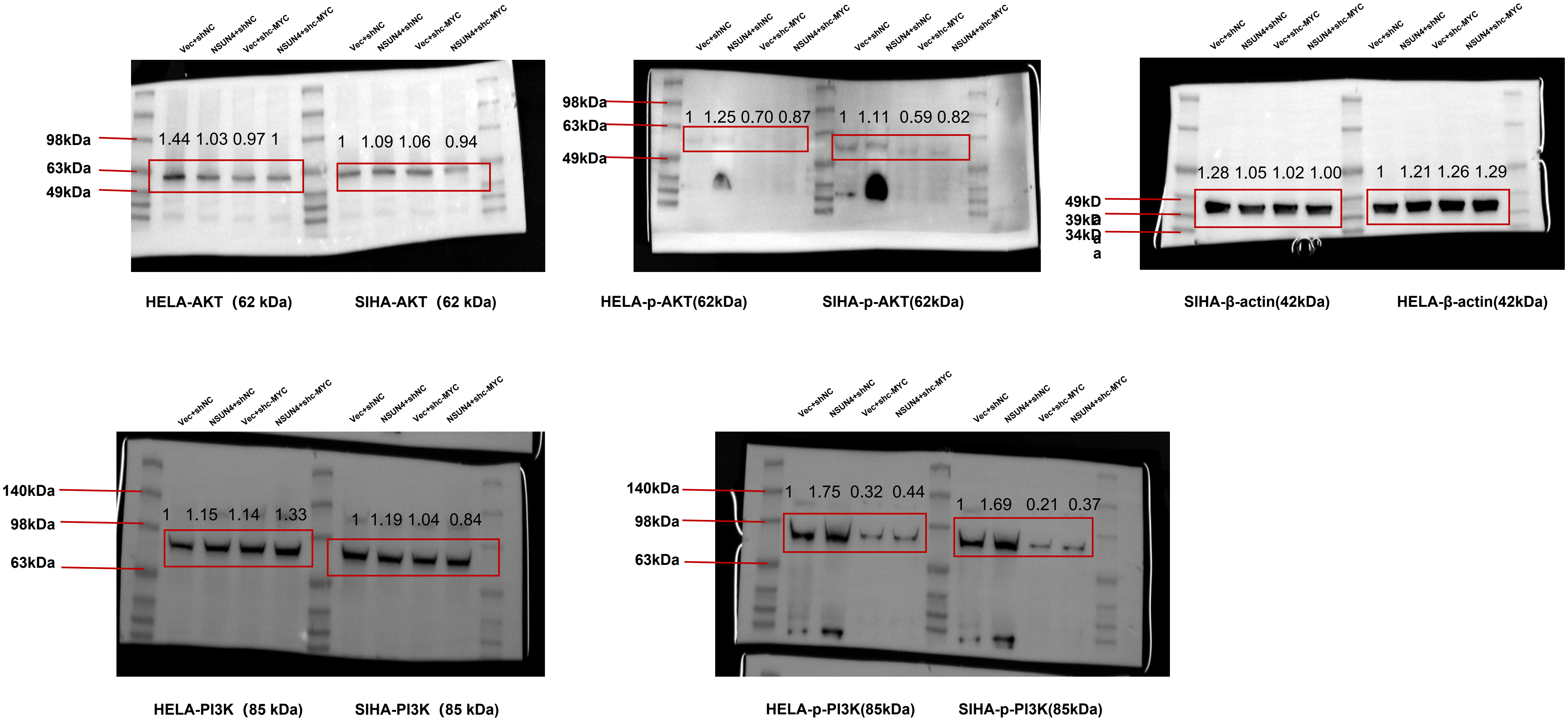

Supplement: Supplementary file 1 [file cancers-18-01392-s001.zip › Figure S1 Original Western blot images corre-sponding to the main figures, including uncropped blot bands, molecular weight markers, and indication of.pdf]
